# Supplementary material for: Genetically stable poliovirus vectors activate dendritic cells and prime antitumor CD8 T cell immunity
Source: Nat Commun. 2020 Jan 27;11:524. doi: 10.1038/s41467-019-13939-z (PMC6985231; doi:10.1038/s41467-019-13939-z)
Supplement: Supplementary file 1 — Supplementary Information [file 41467_2019_13939_MOESM1_ESM.pdf]

Supplementary Information

Genetically Stable Poliovirus Vectors Activate Dendritic Cells and Prime Antitumor CD8 T Cell Immunity

Mosaheb et al.

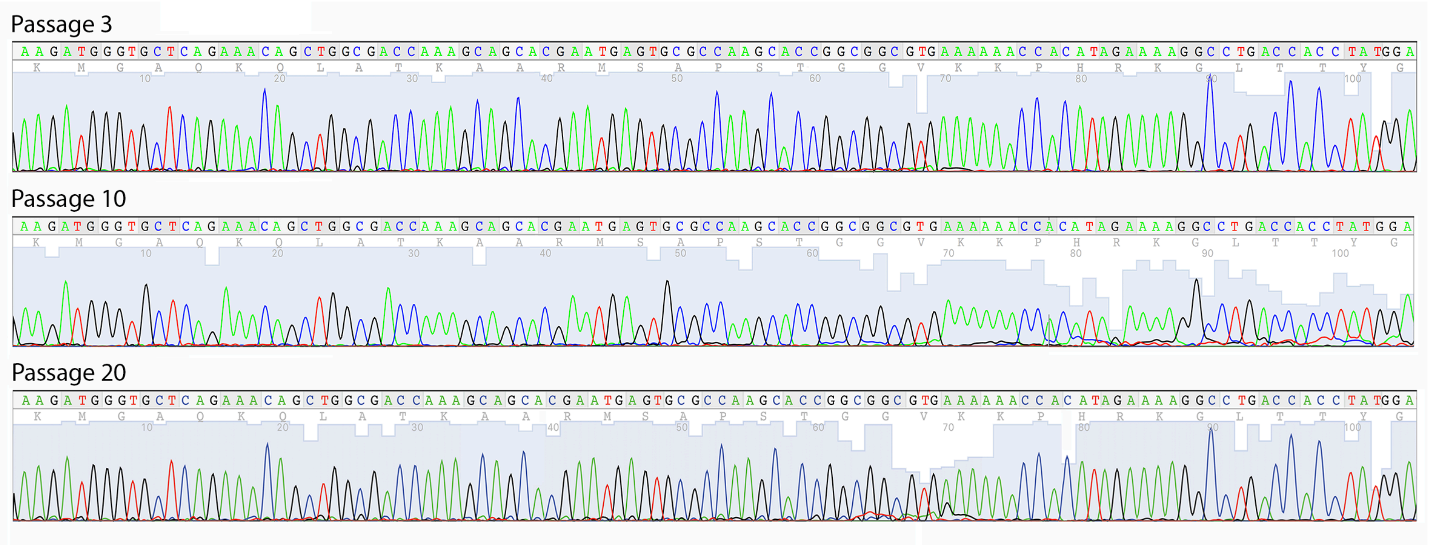

Supplementary Figure 1 (related to Fig. 1g). Sequence chromatogram of RIPO(H3.3) serial passaging.

Sequence chromatograms of RIPO(H3.3) genomic sequences encompassing the entire foreign insert (see **Figure 1g**) obtained from total RNA isolated from infected HeLa cells after 3, 10, or 20 passages. The foreign insert remained unchanged throughout 20 passages.

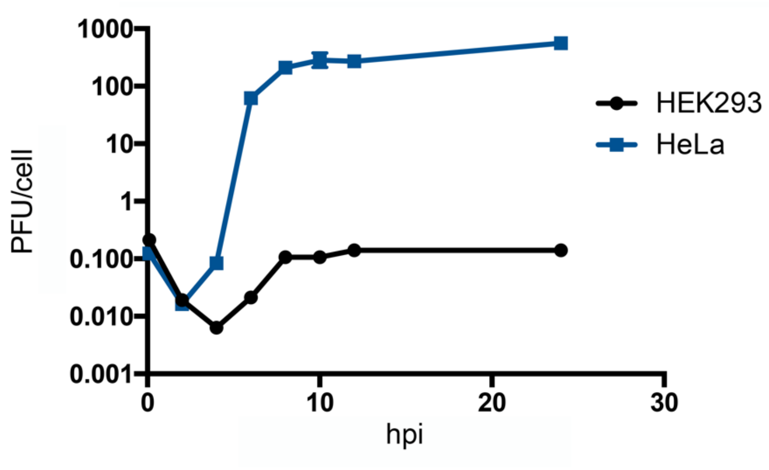

Supplementary Figure 2 (related to Fig. 1h). One-step growth curves of RIPO(H3.3) in HEK293 cells compared to HeLa cells.

The assay was performed as described previously for the PVSRIPO parent.[1] The one-step growth curve of RIPO(H3.3) in HeLa cells is identical to the data shown in **Figure 1h**. RIPO(H3.3) is replication-deficient in HEK293 cells, indicating that it retains the neuron-incompetent phenotype of the HRV2 IRES.[1]

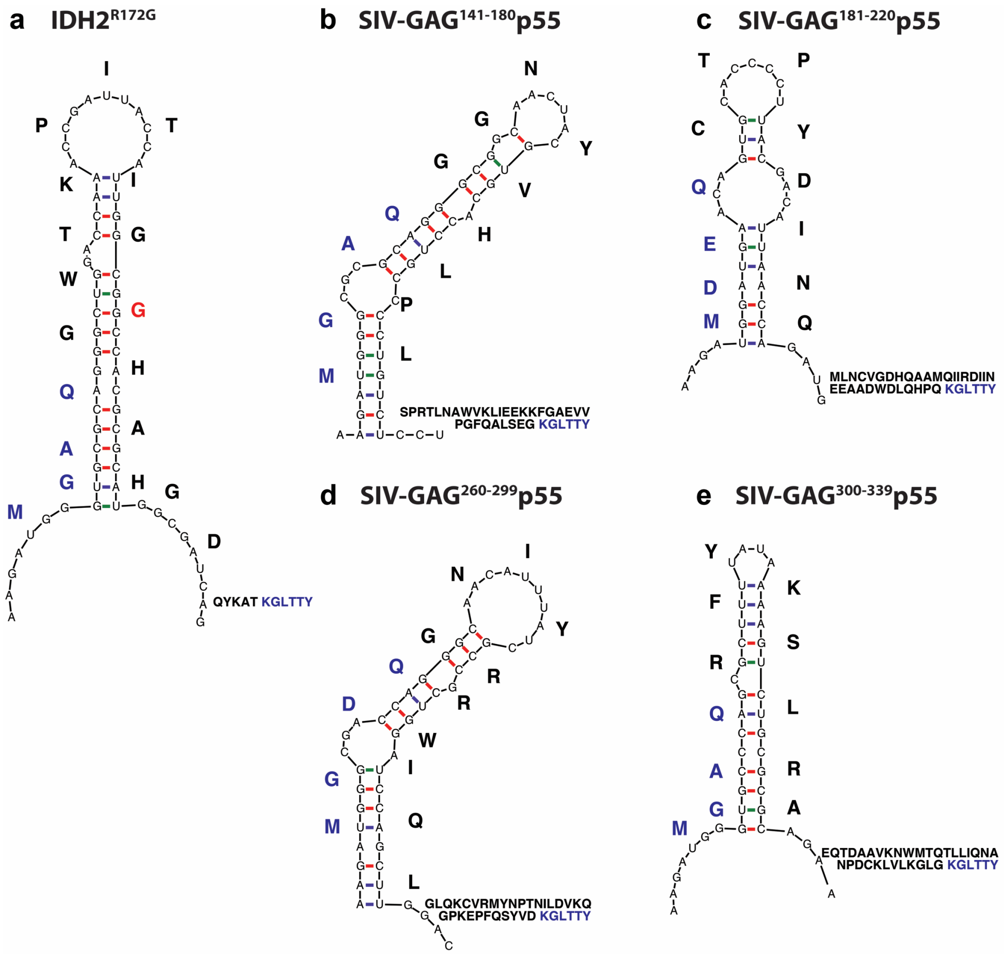

Supplementary Figure 3 (related to Fig. 1). Additional vector designs with confirmed genetic stability.

Vectors delivering a peptide containing the isocitrate dehydrogenase 2 R172G (IDH2^{R172G}) mutation of malignant gliomas (**a**), or peptides corresponding to various segments of the simian immunodeficiency (SIV) p55 Gag antigen (**b**-**e**) were derived and confirmed genetically stable according to the criteria laid out in **Figure 1** and related text. The constructs were derived with primers listed in **Supplementary Table 1**.

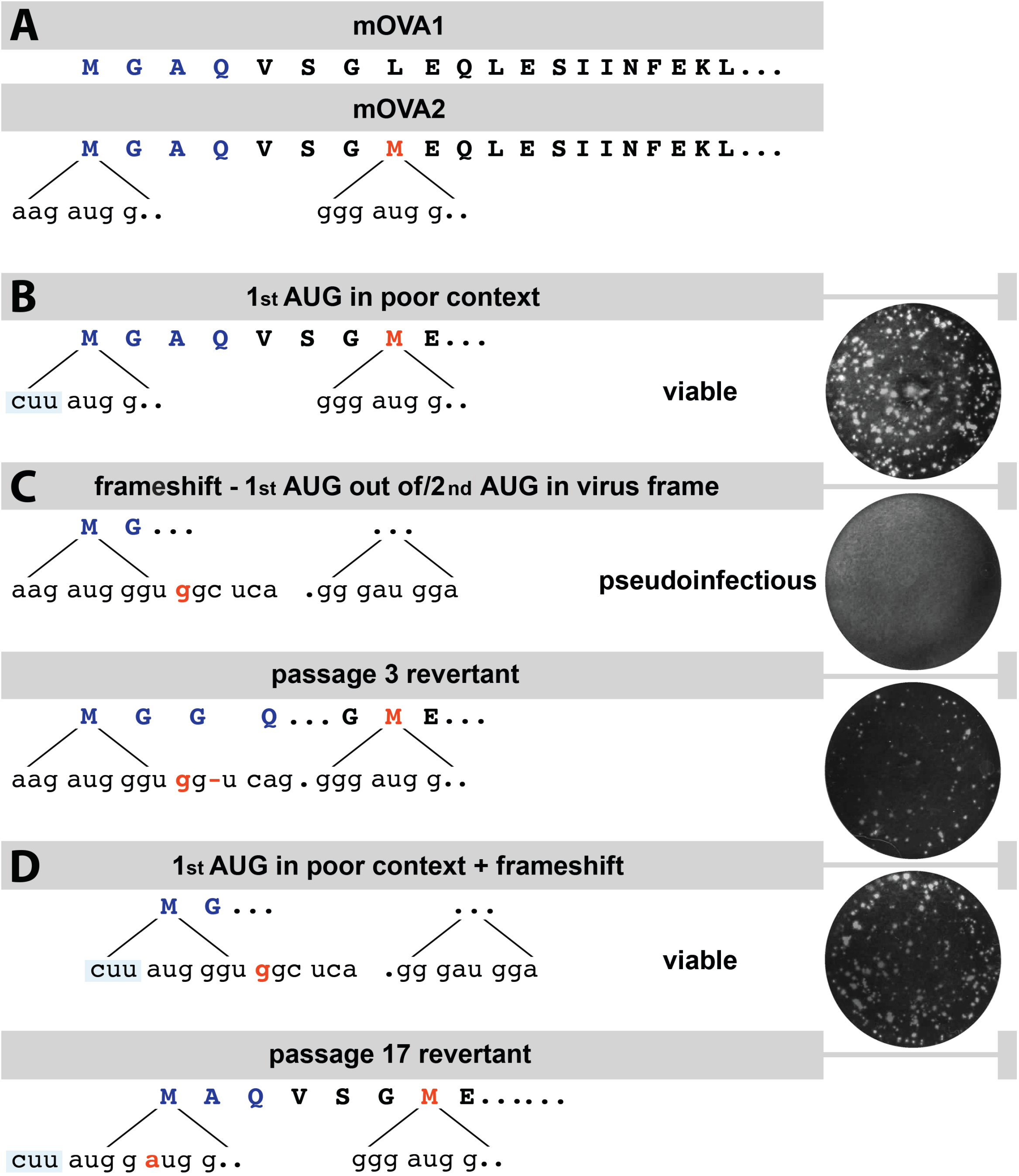
A
mOVA1
M G A Q V S G L E Q L E S I I N F E K L...
mOVA2
M G A Q V S G M E Q L E S I I N F E K L...
aag aug g..
ggg aug g..
B
1st AUG in poor context
M G A Q V S G M E...
cuu aug g..
ggg aug g..
viable
C
frameshift - 1st AUG out of/2nd AUG in virus frame
M G ...
...
aag aug ggu ggc uca
.gg gau gga
pseudoinfectious
passage 3 revertant
M G G Q ... G M E...
aag aug ggu gg-u cag . ggg aug g..
D
1st AUG in poor context + frameshift
M G ...
...
cuu aug ggu ggc uca
.gg gau gga
viable
passage 17 revertant
M A Q V S G M E......
cuu aug g aug g..
ggg aug g..

Supplementary Figure 4 (related to Figs. 1, 2). Initiation codon usage with 'IRES SLD VI replacement vectors' – preference for the 1st AUG in optimal KOZAK context.

(**a**) The C609A mOVA2 adaptation yields two tandem AUGs in optimal KOZAK context (G/A . . **AUG** G). (**b**) To investigate initiation codon usage in mOVA2, we first placed the upstream AUG in poor (native HRV2) KOZAK context. This yielded viable virus. We assume that, since the cryptic AUG at nt 588 in the HRV2 IRES sequence is not used for initiation, the new downstream AUG in mOVA2 can be used for initiation. (**c**) A frameshift introduced between the 2 AUGs (inserted nt is shown in red) yields a pseudoinfectious construct that gains viability upon restoring the vector ORF from the upstream AUG at nt 588. This suggested that, when the upstream AUG is in ideal KOZAK context, it is always used for initiation. (**d**) Placing the upstream AUG at nt 588 in poor KOZAK context combined with the same frameshift instituted in (**c**) yields a viable vector. However, a revertant emerges, where the upstream AUG is in improved KOZAK context and where the vector ORF from the upstream AUG is restored. This, again, suggests that the conserved AUG at nt 588 is preferred for initiation, if in optimal KOZAK context. Examples for plaque phenotypes of the respective constructs (or their adapted variants) are shown in the right-hand column.

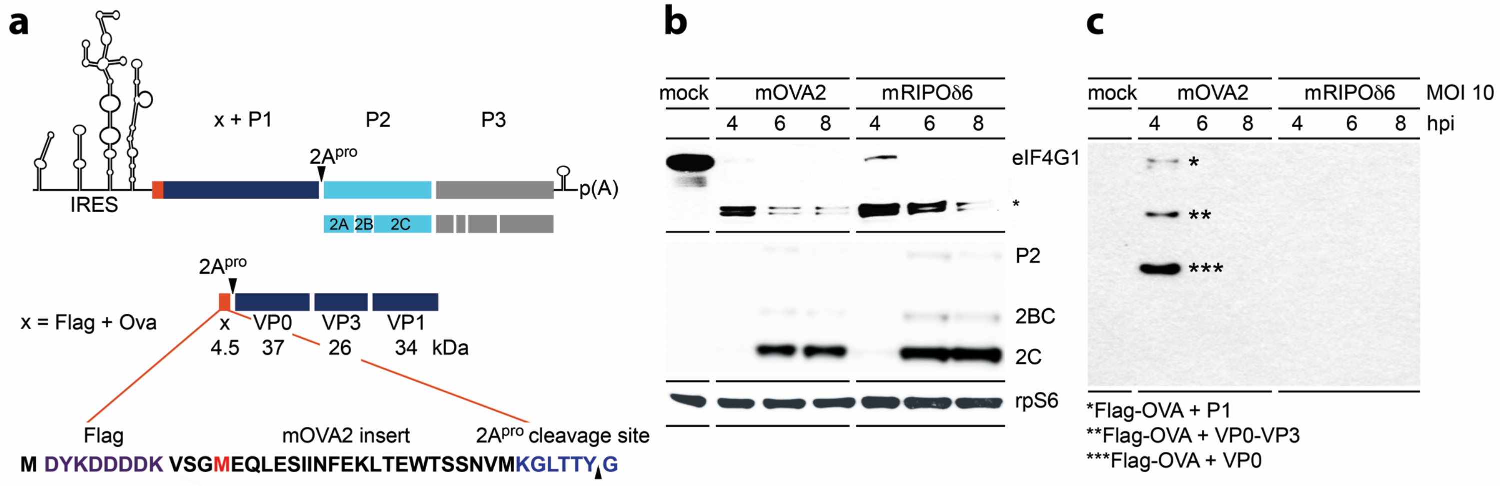

Supplementary Figure 5 (related to Fig. 2). Expression of the SIINFEKL epitope in mOVA2-infected HeLa cells.

(**a**) Since there are no suitable antibodies for detecting the SIINFEKL epitope in immunoblot, we generated a variant of mOVA2 expressing a flag-OVA fusion polypeptide. Processing map of the mOVA2 polyprotein and its P1 (capsid protein-encoding) portion; the flag-OVA antigen is highlighted in red. (**b**, **c**) Immunoblots showing eIF4G1 cleavage and viral translation (b) and flag-OVA immunoprecipitation (c) after infection of HeLa cells with mOVA2 or mRIPOδ6. Flag-OVA epitope fusion polypeptides corresponding to Flag-OVA + P1, Flag-OVA + VP0-VP3 and Flag-OVA + VP0 were detected at 4hpi. Lacking detection at intervals thereafter is likely due to rapid proteolytic processing of the fusion polypeptides.

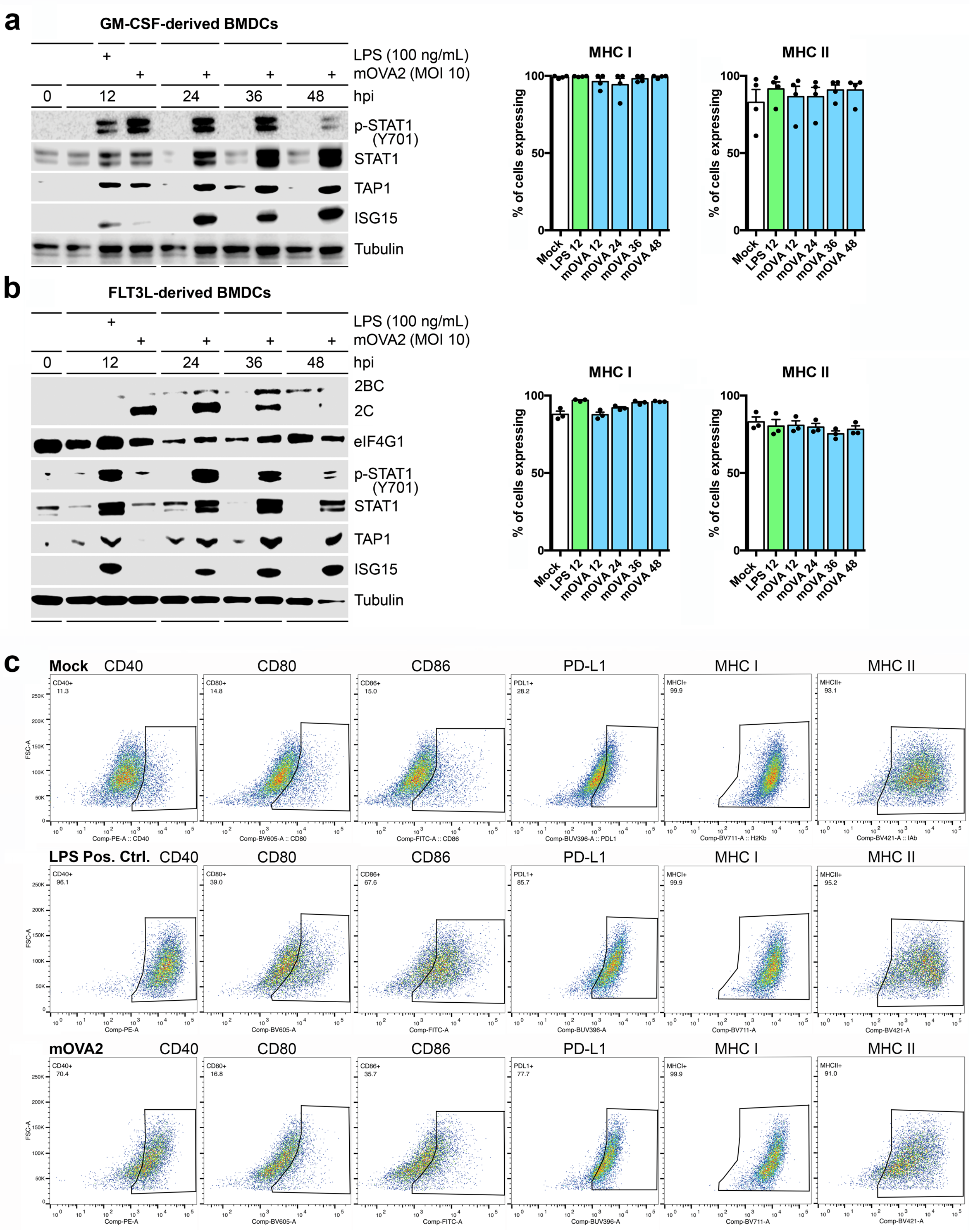
a
GM-CSF-derived BMDCs
LPS (100 ng/mL)
mOVA2 (MOI 10)
hpi
0
12
24
36
48
p-STAT1
(Y701)
STAT1
TAP1
ISG15
Tubulin
MHC I
MHC II
% of cells expressing
Mock
LPS 12
mOVA 12
mOVA 24
mOVA 36
mOVA 48
b
FLT3L-derived BMDCs
2BC
2C
eIF4G1
c
Mock
LPS Pos. Ctrl.
mOVA2
CD40
CD80
CD86
PD-L1
MHC I
MHC II
FSC-A
CD40+
11.3
CD80+
14.8
CD86+
15.0
PDL1+
28.2
MHCI+
99.9
MHCII+
93.1
CD40+
96.1
CD80+
39.0
CD86+
67.6
PDL1+
85.7
MHCI+
99.9
MHCII+
95.2
CD40+
70.4
CD80+
16.8
CD86+
35.7
PDL1+
77.7
MHCI+
99.9
MHCII+
91.0
Comp-PE-A :: CD40
Comp-BV605-A :: CD80
Comp-FITC-A :: CD86
Comp-BUV396-A :: PDL1
Comp-BV711-A :: H2Kb
Comp-BV421-A :: IAb
Comp-PE-A
Comp-BV605-A
Comp-FITC-A
Comp-BUV396-A
Comp-BV711-A
Comp-BV421-A

Supplementary Figure 6 (related to Fig. 3a, b). Immunoblot and flow cytometry analyses of BMDC phenotypes.

(**a**, **b**) (Left panels) Independent, repeat set of immunoblot assays for infected GMCSF- (**Fig. 3a**) and FLT3L-BMDCs (**Fig. 3b**). (Right panels) Surface MHC I/II expression for GMCSF- and FLT3L-BMDCs upon infection with mOVA2 or treatment with LPS. (**c**) Gating strategy for CD40, CD80, CD86, PDL1 and MHC I/II positive BMDCs (Gated on $CD11c^{+}$) in **Figure 3a, b**.

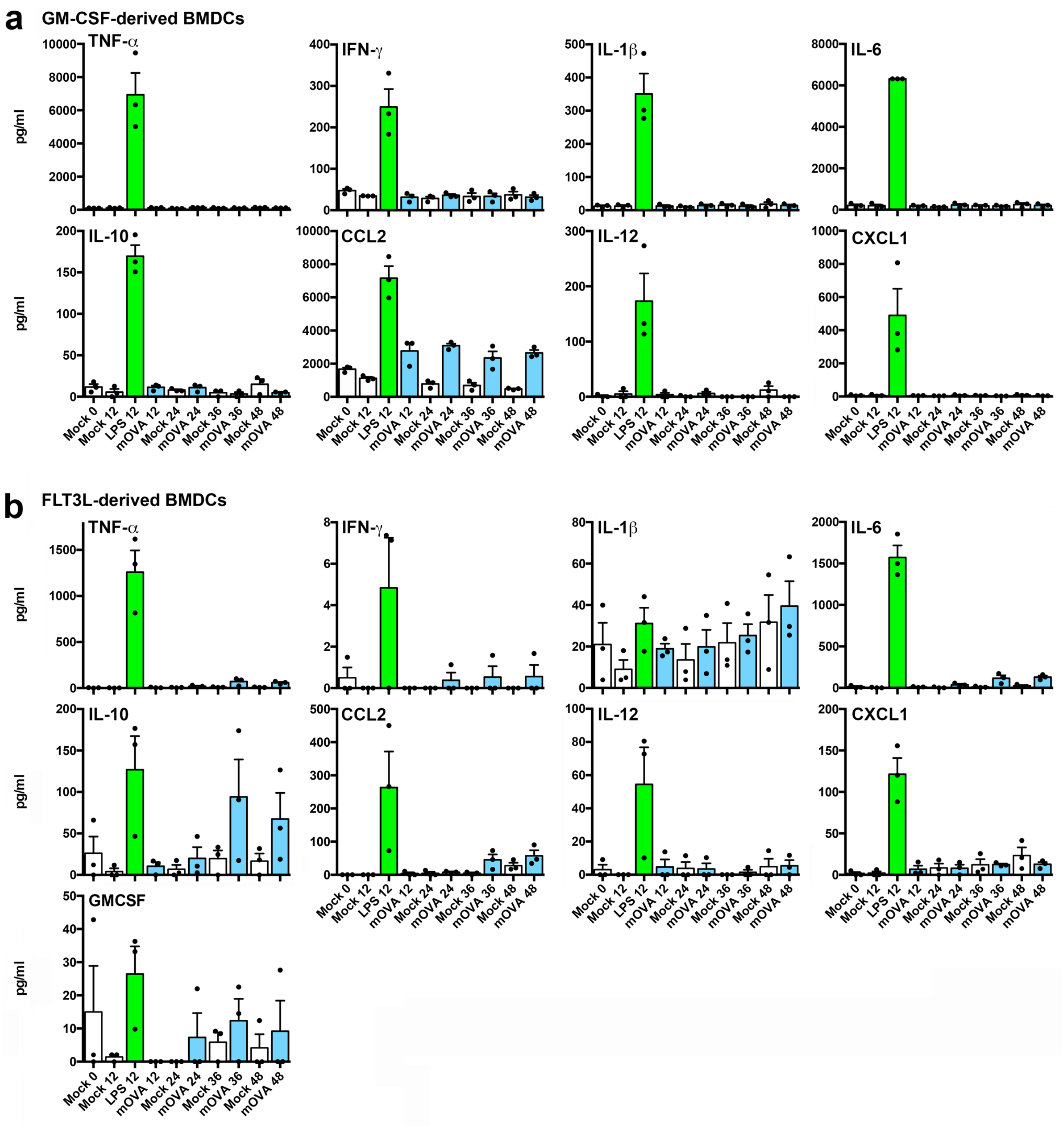
a
GM-CSF-derived BMDCs
TNF-α
IFN-γ
IL-1β
IL-6
IL-10
CCL2
IL-12
CXCL1
pg/ml
Mock 0
Mock 12
LPS 12
mOVA 12
Mock 24
mOVA 24
Mock 36
mOVA 36
Mock 48
mOVA 48
b
FLT3L-derived BMDCs
GMCSF

Supplementary Figure 7 (related to Fig. 3c, d). Cytokine analyses of infected BMDCs.

(**a**, **b**) Cytokine bead array results for the set of cytokines not shown in **Figure 3** in tests of GMCSF- (**a**) or FLT3L- (**b**) BMDCs upon infection with mOVA2 or treatment with LPS (see **Fig. 3c, d** for IFNα, IFNβ, CCL5 and CXCL10). GMCSF is not shown for (**a**) because the medium was supplemented with GMCSF.

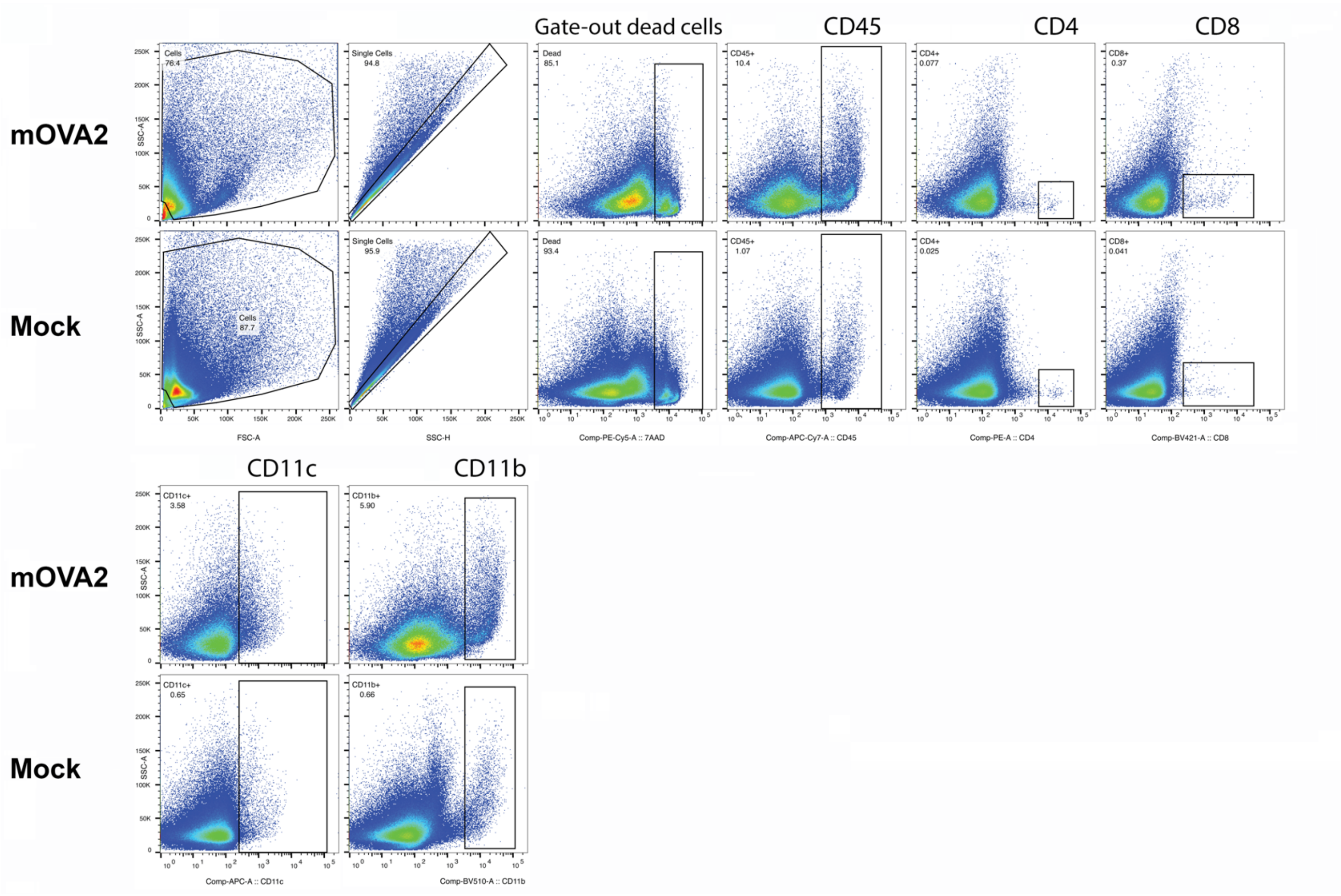

Supplementary Figure 8 (related to Fig. 4b). Gating strategy for analyses of skeletal muscle immune cell infiltrates in immunized hCD155-tg mice.

Gating strategy for skeletal muscle immune cell infiltrates (CD45, CD4, CD8, CD11c and CD11b) after mOVA2 or mock (DMEM) immunization in **Figure 4b**.

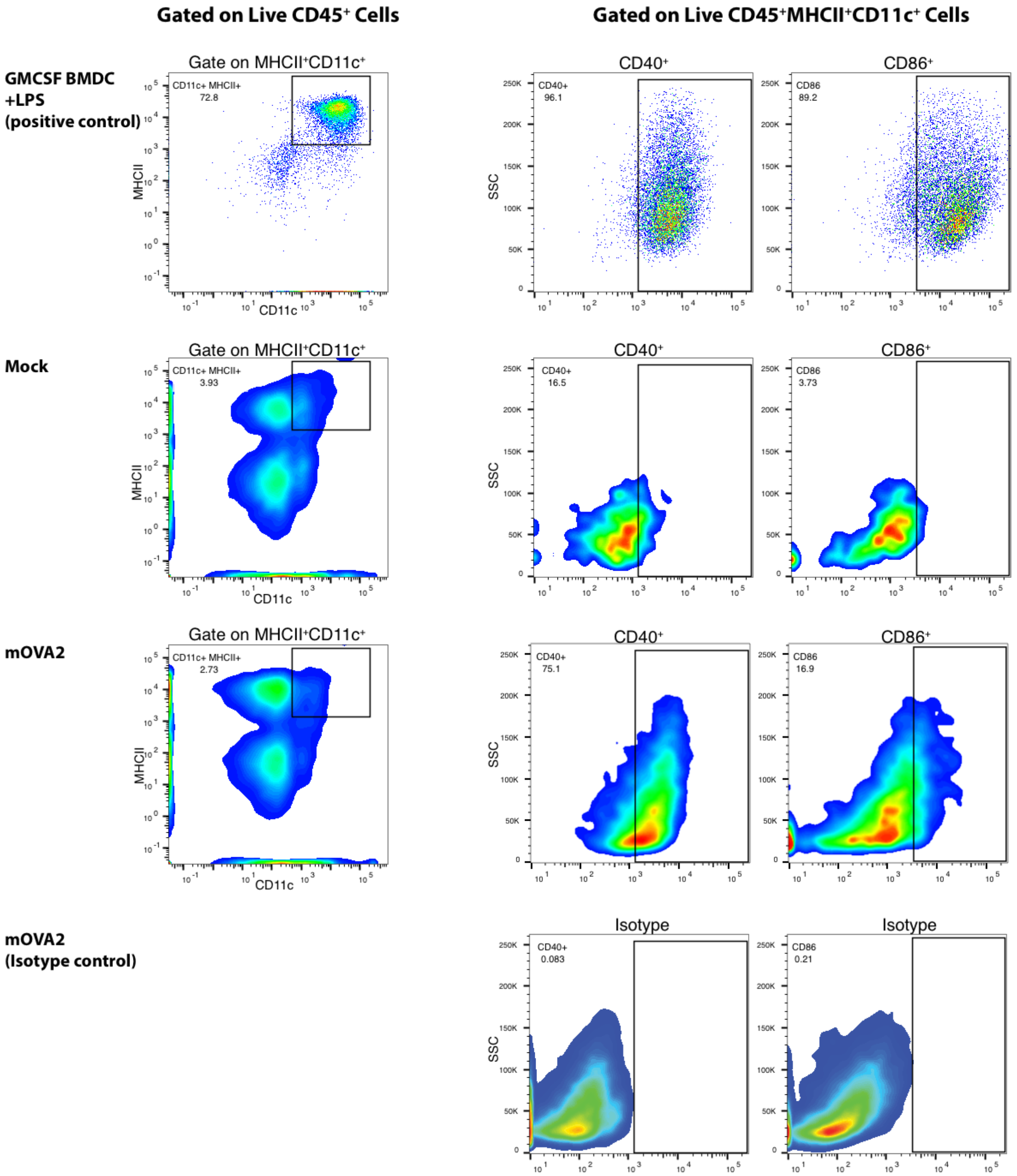

Gated on Live CD45+ Cells
Gated on Live CD45+MHCII+CD11c+ Cells
GMCSF BMDC
+LPS
(positive control)
Mock
mOVA2
mOVA2
(Isotype control)
Gate on MHCII+CD11c+
CD11c+ MHCII+
72.8
3.93
2.73
MHCII
CD11c
CD40+
CD86+
CD40+
96.1
16.5
75.1
0.083
CD86
89.2
3.73
16.9
0.21
Isotype
SSC

Supplementary Figure 9 (related to Fig. 4c). Representative flow cytometry data and gating strategy for analyses of skeletal muscle immune cell infiltrates in immunized hCD155-tg mice.

Gating analysis for CD40 and CD86 positive skeletal muscle-infiltrating DCs (CD11c^{+}MHCII^{+}) in **Figure 4c**.

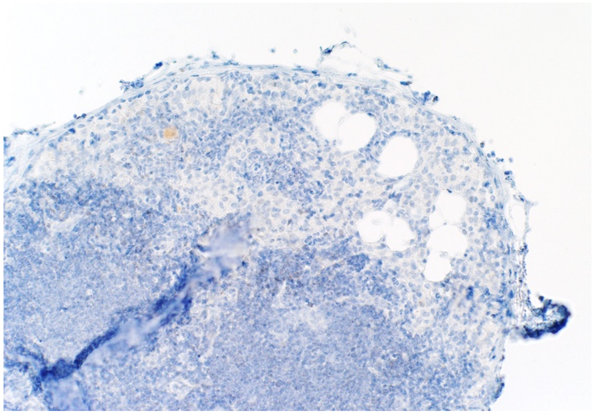

Supplementary Figure 10 (related to Fig. 4d). Immunohistochemical staining for H3.3^{K27M} in a popliteal lymph node of a mock-immunized hCD155-tg mouse.

The assay was conducted as described for **Figure 4d**.

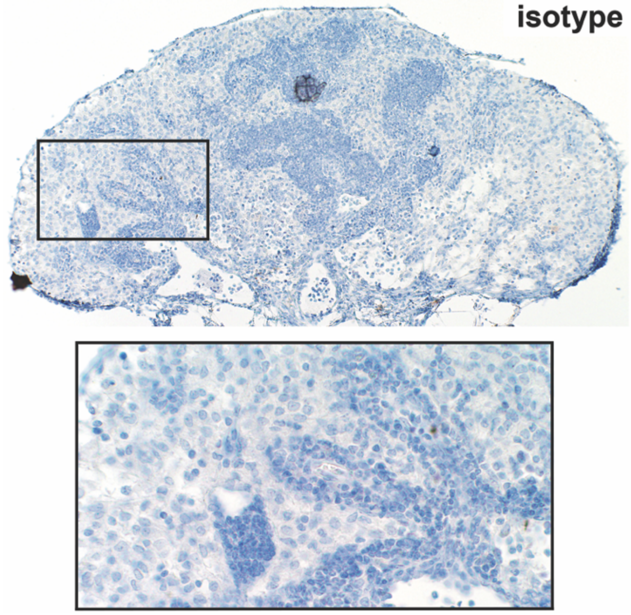

Supplementary Figure 11 (related to Fig. 4d). Immunohistochemical staining with an isotype-matched, non-specific control of a lymph node section consecutive to those shown in Figure 4d.

The assay was conducted as described for **Figure 4d**.

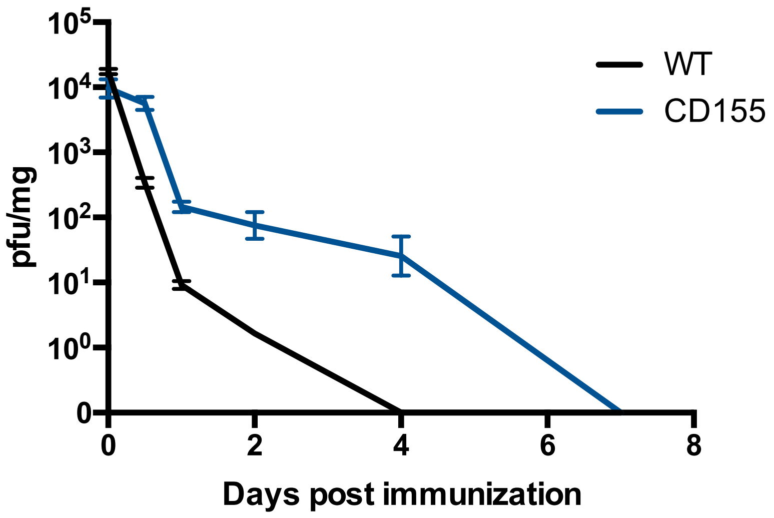

Supplementary Figure 12 (related to Fig. 4). Intramuscular replication of mRIPO(H3.3) in hCD155-tg mice.

Recovery of infectious mRIPO(H3.3) in skeletal muscle tissue, after intramuscular inoculation (10^7 pfu) in wild type or hCD155-tg mice, was assessed over time using previously reported procedures for an identical assay.[2]

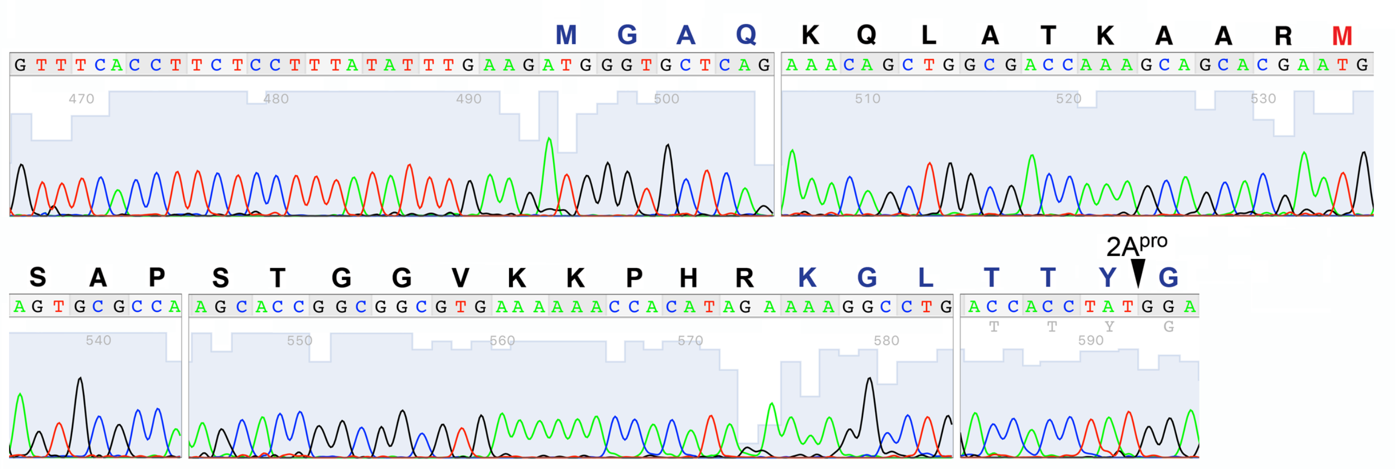

Supplementary Figure 13 (related to Fig. 4). Genetic stability of mRIPO(H3.3) after intramuscular replication in hCD155-tg mice.

Chromatogram of sequences obtained from mRIPO(H3.3) recovered from skeletal muscle of hCD155-tg mice 4 days after intramuscular inoculation (10^7 pfu). The region encompassing the engineered initiation codon, the coding region for the H3.3^{K27M} epitope and the engineered 2A^{pro} cleavage site are indicated. Compare to the original vector design (**Fig. 1g**) and to sequence chromatograms after serial passage in HeLa cells (**Supplementary Fig. 1**).

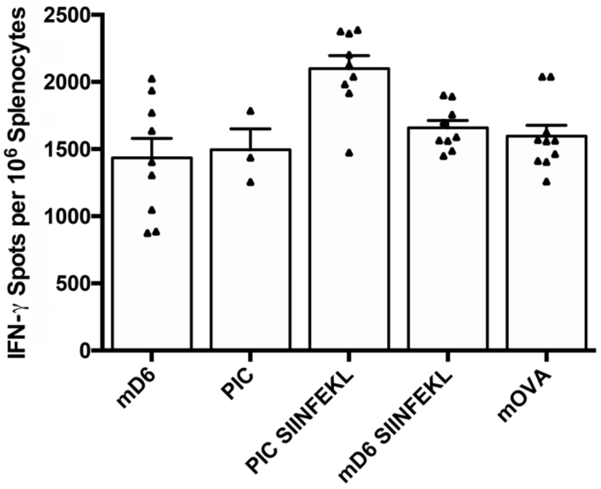

Supplementary Figure 14 (related to Fig. 5a, b). Positive control for IFN-γ ELISPOT assay.

Splenocytes from **Figure 5a, b** were treated with Concanavalin A as a positive control for IFN-γ ELISPOT assay. See details for the assay in **Figure 5a, b** and related text.

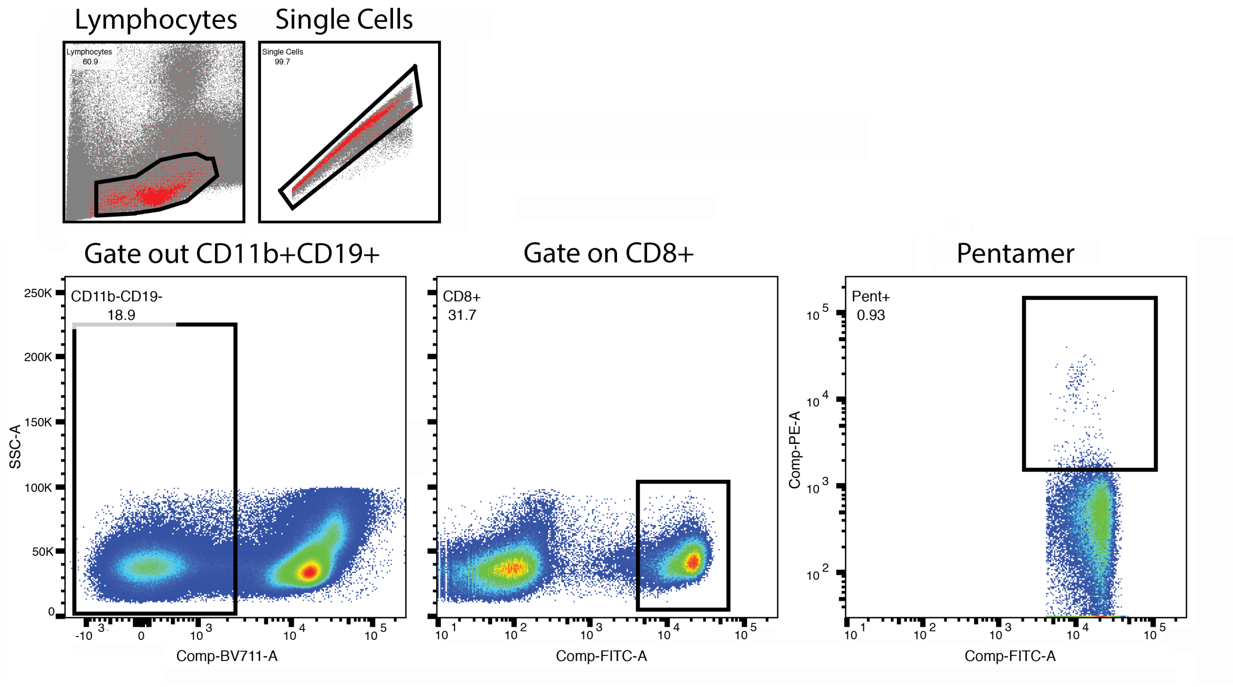

Supplementary Figure 15 (related to Fig. 5c, d). Gating strategy for H2Kb-SIINFEKL pentamer and HLA-A2-RMSAPSTGGV tetramer staining of peripheral CD8 T cells.

Representative sample shown is H2Kb-SIINFEKL pentamer staining of an mOVA2-immunized mouse.

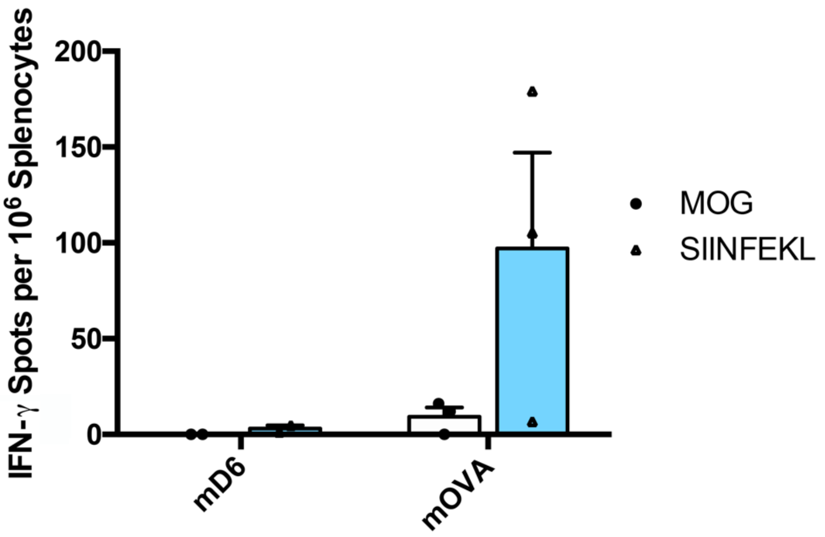

Supplementary Figure 16 (related to Fig. 5c, d). Immunization with mOVA2-infected DCs induces SIINFEKL-specific CD8 T cell responses.

FLT3L-BMDCs were generated from hCD155-tg mice, as described for **Figure 3**. The cells were infected with mRIPOδ6 or mOVA2 (MOI 10) (2h). The infected BMDCs were washed in PBS three times and inoculated intraperitoneally into wild type C57Bl6 mice (not expressing hCD155 and, thus, unable to sustain poliovirus replication in any compartment). An identical booster dose was administered 2 weeks after prime and an IFN-γ ELISPOT assay was performed seven days later.

a Gating Strategy for CD45+CD3+ cells

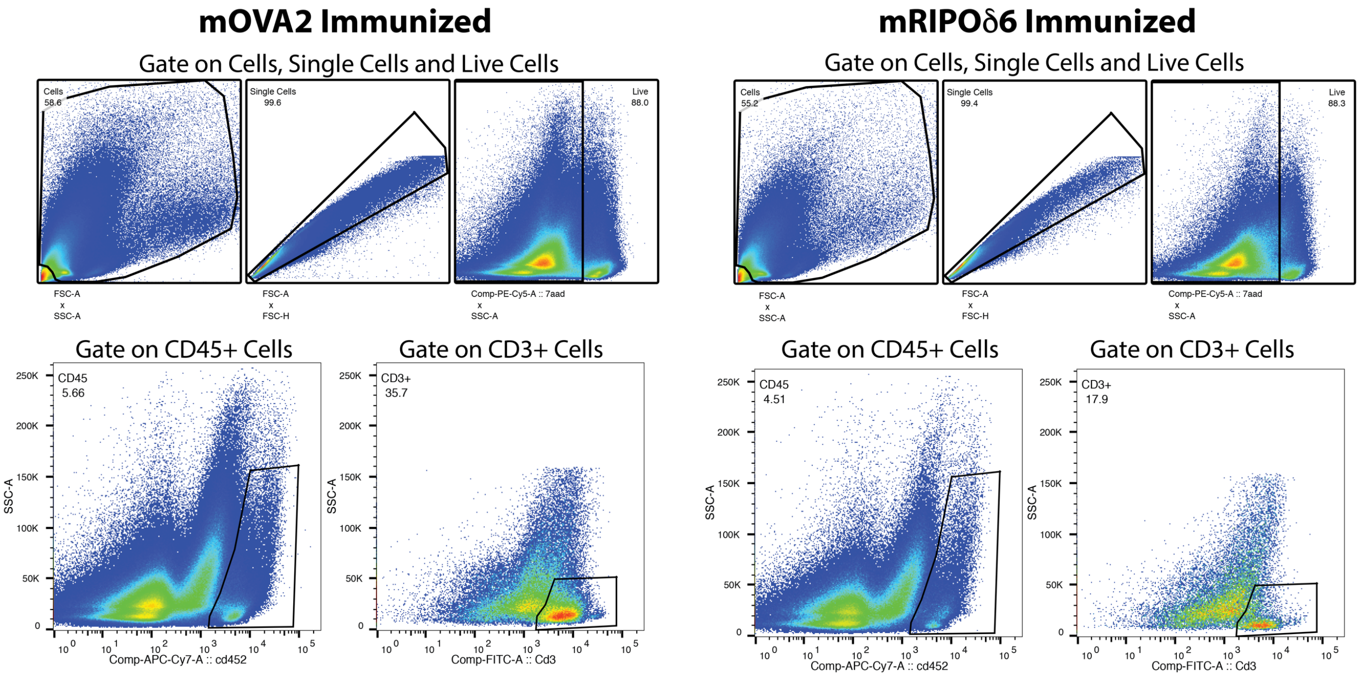

b Gating Strategy for CD8+ and CD4+ cells

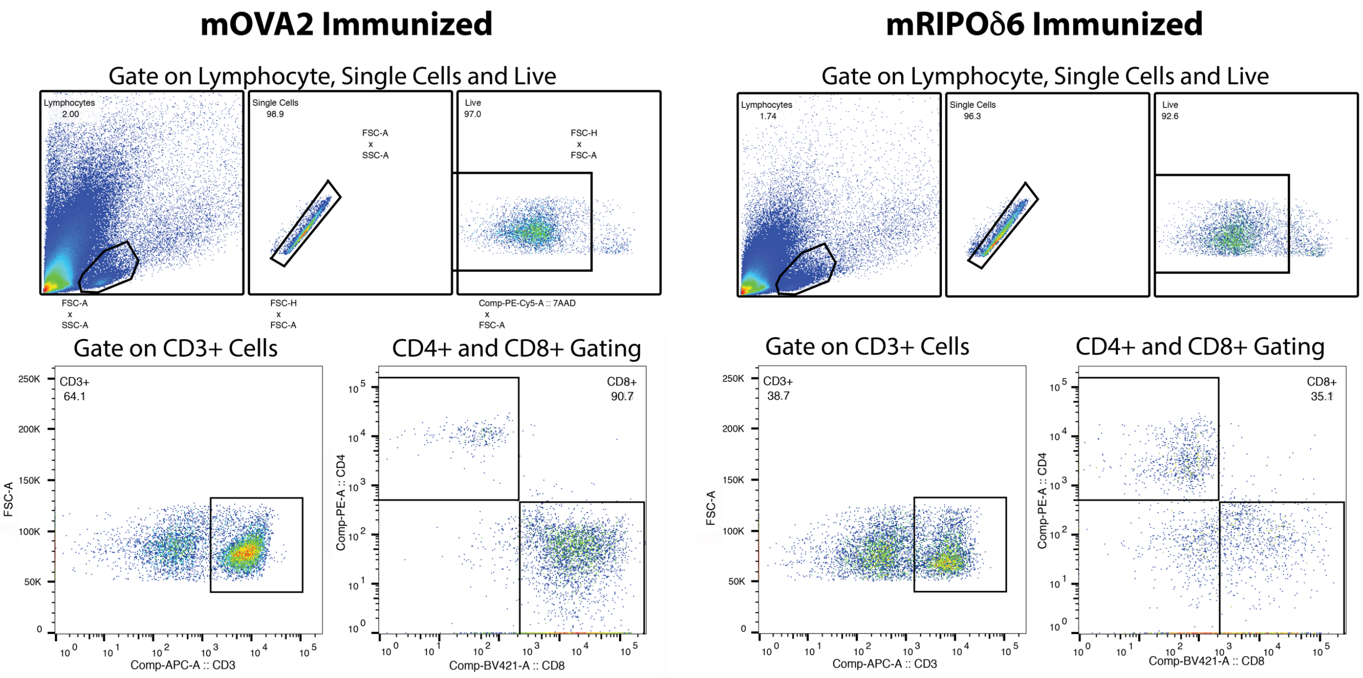

Supplementary Figure 17 (related to Fig 6c). Representative flow cytometry data and gating strategy for tumor-infiltrating lymphocytes.

(**a**) Gating strategy for testing tumor-infiltrating CD45+ and CD3+ cells. (**b**) Gating strategy for assessing the CD8+:CD4+ ratio.

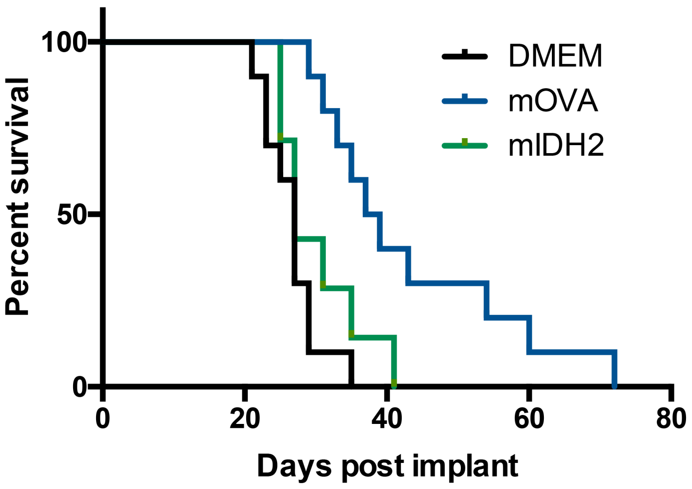

Supplementary Figure 18 (related to Fig 6).

Results of a replicate assay of mOVA2 immunization of hCD155-tg mice bearing B16-OVA tumor implants. Experimental groups include mOVA2 immunized- (n=10), mIDH2 immunized- (see **Supplementary Figure 3a**) (n=7), and mock (DMEM)-immunized animals (n=10). Mice were immunized on day 0, boosted on day 14, and tumors were implanted on day 21.

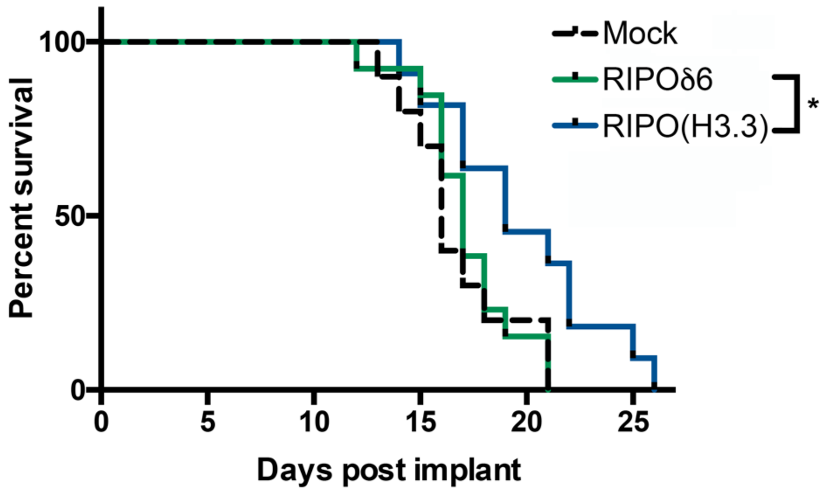

Supplementary Figure 19 (related to Fig 7).

Results of a replicate assay showing that mRIPO(H3.3) immunization extends survival in an immunocompetent, intracerebral malignant glioma model. The assay was conducted as described for Figure 7e. mRIPO(H3.3)-immunized mice survived significantly longer than mRIPOδ6-immunized animals [P=0.0414, Log-rank (Mantel-Cox) test, median survival; mRIPOδ6 n=13, mRIPO(H3.3) n=11]; Mock group (n=10) included for visual illustration only. Data from two independent experiments combined.

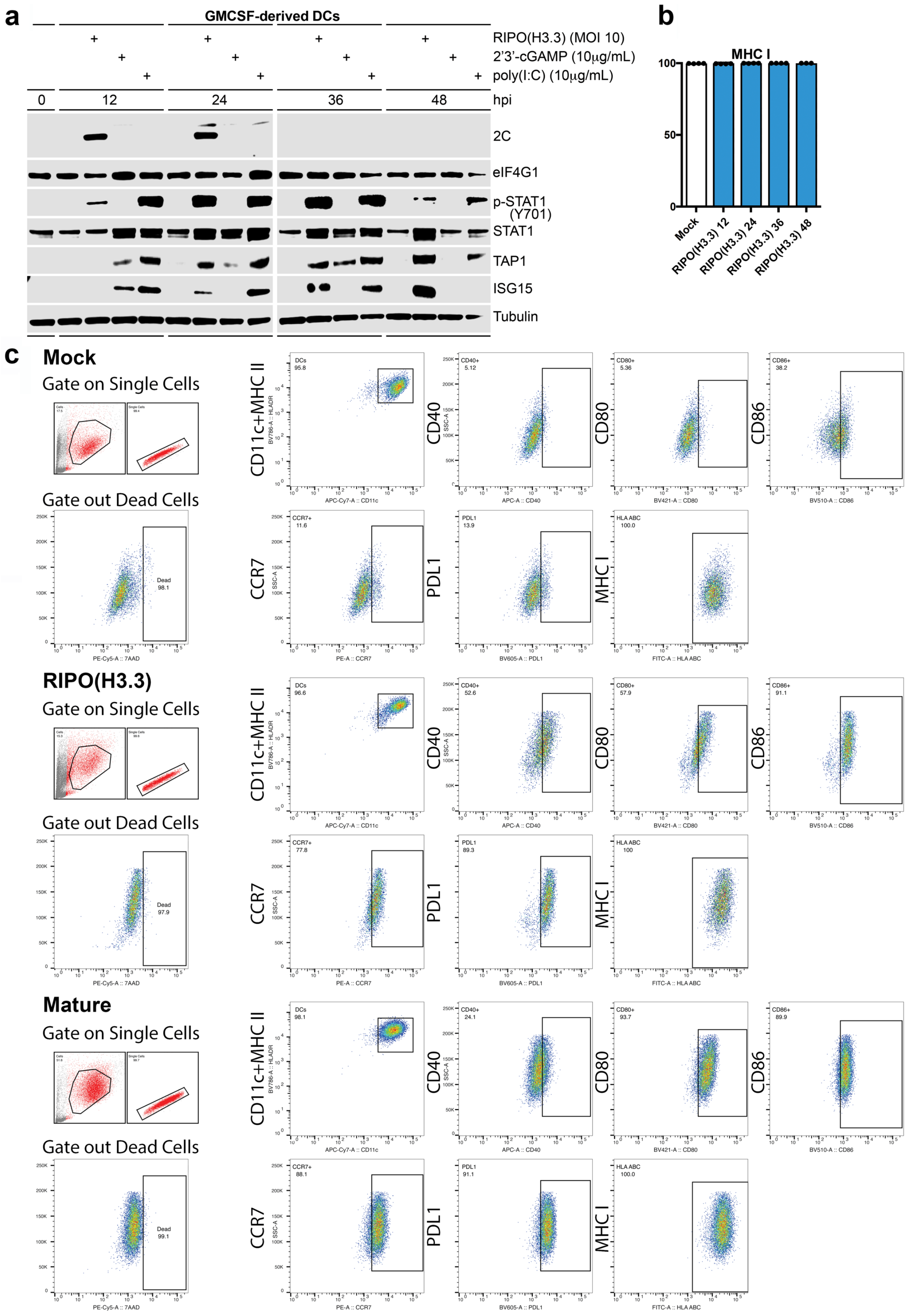
a
GMCSF-derived DCs
RIPO(H3.3) (MOI 10)
2'3'-cGAMP (10µg/mL)
poly(I:C) (10µg/mL)
0
12
24
36
48
hpi
2C
eIF4G1
p-STAT1 (Y701)
STAT1
TAP1
ISG15
Tubulin
b
MHC I
100
50
0
Mock
RIPO(H3.3) 12
RIPO(H3.3) 24
RIPO(H3.3) 36
RIPO(H3.3) 48
c
Mock
Gate on Single Cells
Gate out Dead Cells
RIPO(H3.3)
Gate on Single Cells
Gate out Dead Cells
Mature
Gate on Single Cells
Gate out Dead Cells
CD11c+MHC II
CD40
CD80
CD86
CCR7
PDL1
MHC I

Supplementary Figure 20 (related to Fig. 8a and c). Immunoblot, flow cytometry analyses, and gating strategy of infected human DC phenotypes.

(**a**) Repeat immunoblot assay of human GMCSF-derived DCs after infection with RIPO(H3.3) (compare to **Fig. 7a**). (**b**) Surface MHC I expression on infected DCs. (**c**) Gating strategy for DC phenotype in **Figure 7c**. Mature DCs, DCs treated with maturation cocktail as previously described[3, 4] are included as positive control.

GM-CSF-derived DCs

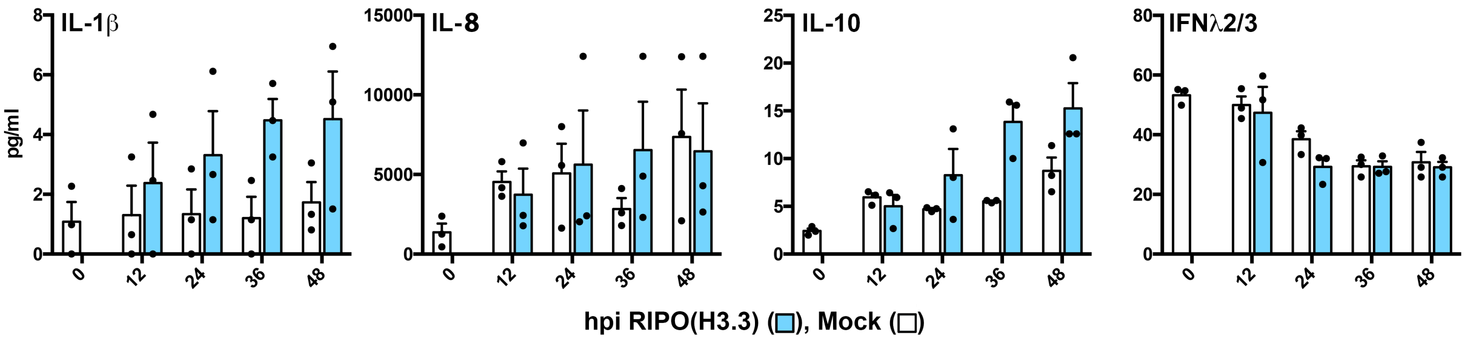

Supplementary Figure 21 (related to Fig. 8d). Cytokine analyses of infected human DCs.

Cytokine bead array results for the set of cytokines not shown in **Figure 7d** (tests from RIPO(H3.3)-infected human DCs at 0, 12, 24, 36 and 48 hours post infection, hpi).

Supplementary Table S1. Primers used in this study.

Primer[a]	Sequence (5’ to 3’)
mOVA1-5’	cctttatatttgaagatgggggctcaggtcagtgggctggagcaactggagagcatcatcaactttgagaaactgactgaatgg
mOVA1-3’	ctgagctccataggtggtcagacctttcatcacgttactactagtccattcagtcagtttctcaaagttg
mOVA2-5’	cctttatatttgaagatgggggctcaggtcagtggg**a**tggagcaactggagagcatcatcaactttgagaaactgactgaatgg
mOVA2-PC-5’	cctttatatttgcttatgggtgctcaggtcagtgggatggagc
mOVA2-PC-3’	ccataagcaaatataaaggagaa
mOVA-flag-5’	cctttatatttgaagatgggtggctcaggtcagtgggatggagc
mOVA2-FS-5’	cctttatatttgaagatggattacaaggatgacgacgataaggtcagtgggatggagcca
RIPO(H3.3)-5’	cctttatatttgaagatgggtgctcagaaacagctggcgaccaaagcagcacgaatgagtgc
RIPO(H3.3)-3’	cctgagctccataggtggtcaggccttttctatgtggtttttcacgccgccggtgcttggcgcactcattcg
hIRES-5’	aggaattcaacttagaagtttttcaca
hIRES-3’	gcacccatcttcaaatataaaggagaaggtgaaacacg
mIRES-5’	cccacgtggcggctagtactcc
mIRES-3’	cccatcttcaaatataaaggagaaggtgaaacacg
Seq-3’	aatggtagaaccaccatacgc
IDH2(R172G)-5’	cctttatatttgaagatgggtgcgcagggctggaccaaaccgattaccattggcggccacgcgcatggc
IDH2(R172G)-3’	ctgagctccataggtggtcaggcctttggtcgctttatactgatcgccatgcgcgtg
SIV139.1-5’	cttttatatttgaagatgggcgcgcagggcggcaactacgtgcacctgcccctgtctcctagaacactgaacgcatgggtg
SIV139.1-3’	ctgagctccataggtggtcagtcctttgccttcgctcagagcttgaaatccaggcaccacttcagcgccaaatttttttcttcaattaatttcacccatgcgttcagtg
SIV139.2-5’	ctttttatttgaagatggatgaacagtgcaccccttacgacattaaccagatgctaaactgcgtgggcgaccatcaggctgctatgcaaatc
SIV139.2-3’	ctgagctccataggtggtcagtcctccataggtggtcaggcctttctgaggatgctgcagatcccaatcagcggcttcttcgttaataatatctctaatgatttgcatagcagcctg
SIV259.1-5’	cttttatatttgaagatgggcgaccagggcaacatttatcgccgctggatccagcttggacttcagaagtgcgtgcgcatgtataaccccaccaacatcctgg
SIV259.1-3’	ctgagctccataggtggtcagtcctccataggtggtcaggcctttatccacatagctctgaaagggttcttttgggccctgtttcacatccaggatgttggtgggg
SIV259.2-5’	cttttatatttgaagatgggtgcccagcgcttttataaaagtctgcgcgcagaacagactgatgctgctgtgaaaaactggatgacccagaccctgctgattcagaacgccaaccc
SIV259.2-3’	ctgagctccataggtggtcagtcctccataggtggtcaggcctttgcccaggcctttcagcaccagtttgcaatcagggttggcgttctgaatcagc

[a]Primer nomenclature (see Materials and Methods for primer use in vector cloning and sequencing).

Supplementary Table S2

Immunoblot antibodies

Antibody	Company	Item Number (dilution)
eIF4G1	Cell Signaling Technologies	2469 (1:1,000)
STAT1	Cell Signaling Technologies	9172 (1:1,000)
STAT1P (Y701)	Cell Signaling Technologies	9167 (1:1,000)
TAP1	Cell Signaling Technologies	12341 (1:1,000)
ISG 15	Cell Signaling Technologies	2743, 2758 (1:1,000)
Tubulin	Sigma-Aldrich	T9026 (1:10,000)
2C (Viral Protein)	Proprietary	N/A (1:1,000)
H3.3K27M	Cell Signaling Technologies	74829 (WB 1:1,000) (IHC 1:1,600)
S6 Ribosomal Protein	Cell Signaling Technologies	2217 (1:1,000)
GAPDH	Cell Signaling Technologies	5174 (1:1,000)
Flag	Cell Signaling Technologies	2368 (1:1,000)

Flow cytometry antibodies

Antibody	Conjugate	Company	Item Number
CD11c	APC	Biolegend	117310 (1:100)
CD80	BV605	Biolegend	104729 (1:100)
CD86	Alexa Fluor 488	Biolegend	105017 (1:100)
CD40	PE	Biolegend	124609 (1:100)
H2Kb	BV711	Biolegend	742862 (1:100)
H2IAb	BV421	Biolegend	107631 (1:100)
PDL1	BUV395	BD	745616 (1:100)
7AAD	N/A	Biolegend	420403 (1:100)
CD45.2	APC	Biolegend	109814 (1:100)
CD8	BV421	Biolegend	100738 (1:100)
CD11b	BV711	Biolegend	101241 (1:100)
CD19	BV711	Biolegend	115555 (1:100)
CD4	PE	Biolegend	100512 (1:100)
SIINFEKL Pentamer	PE	Pro-immune	N/A (1:20)
CD8 (KT15)	FITC	ThermoFisher	MA5-16761 (1:100)
TruStain FcX	N/A	Biolegend	101320 (1:50)
Human TruStain FcX	N/A	Biolegend	422301 (1:50)
H3.3K27M Tetramer	PE	NIH Tetramer Core	N/A (1:20)
Human CD11c	APC-Cy7	Biolegend	337218 (1:100)
Human HLADR	BV786	Biolegend	307642 (1:100)
Human CD40	APC	Biolegend	334310 (1:100)
Human CD80	BV421	Biolegend	305222 (1:100)
Human CD86	BV510	Biolegend	305432 (1:100)
Human CCR7	PE	Biolegend	353204 (1:100)
Human HLA ABC	FITC	Biolegend	311403 (1:100)
Human PDL1	BV605	Biolegend	329724 (1:100)

References

1. Campbell, S.A., Lin, J., Dobrikova, E.Y. & Gromeier, M. Genetic determinants of cell type-specific poliovirus propagation in HEK 293 cells. *J Virol* **79**, 6281-6290 (2005).
2. Gromeier, M. & Wimmer, E. Mechanism of injury-provoked poliomyelitis. *Journal of virology* **72**, 5056-5060 (1998).
3. Brown, M.C. *et al.* Cancer immunotherapy with recombinant poliovirus induces IFN-dominant activation of dendritic cells and tumor antigen-specific CTLs. *Sci Transl Med* **9** (2017).
4. Nair, S., Archer, G.E. & Tedder, T.F. Isolation and generation of human dendritic cells. *Curr Protoc Immunol* **Chapter 7**, Unit7 32 (2012).
