# Supplementary material for: Genetically stable poliovirus vectors activate dendritic cells and prime antitumor CD8 T cell immunity
Source: Nat Commun. 2020 Jan 27;11:524. doi: 10.1038/s41467-019-13939-z (PMC6985231; doi:10.1038/s41467-019-13939-z)
Supplement: Supplementary file 3 — Reporting Summary [file 41467_2019_13939_MOESM3_ESM.pdf]

nature research

Corresponding author(s): Matthias Gromeier
Last updated by author(s): Dec 4, 2019

Reporting Summary

Nature Research wishes to improve the reproducibility of the work that we publish. This form provides structure for consistency and transparency in reporting. For further information on Nature Research policies, see Authors & Referees and the Editorial Policy Checklist.

Statistics

For all statistical analyses, confirm that the following items are present in the figure legend, table legend, main text, or Methods section.

n/a	Confirmed	
☐	☒	The exact sample size (*n*) for each experimental group/condition, given as a discrete number and unit of measurement
☐	☒	A statement on whether measurements were taken from distinct samples or whether the same sample was measured repeatedly
☐	☒	The statistical test(s) used AND whether they are one- or two-sided<br>*Only common tests should be described solely by name; describe more complex techniques in the Methods section.*
☐	☒	A description of all covariates tested
☒	☐	A description of any assumptions or corrections, such as tests of normality and adjustment for multiple comparisons
☐	☒	A full description of the statistical parameters including central tendency (e.g. means) or other basic estimates (e.g. regression coefficient) AND variation (e.g. standard deviation) or associated estimates of uncertainty (e.g. confidence intervals)
☐	☒	For null hypothesis testing, the test statistic (e.g. *F*, *t*, *r*) with confidence intervals, effect sizes, degrees of freedom and *P* value noted<br>*Give P values as exact values whenever suitable.*
☒	☐	For Bayesian analysis, information on the choice of priors and Markov chain Monte Carlo settings
☒	☐	For hierarchical and complex designs, identification of the appropriate level for tests and full reporting of outcomes
☒	☐	Estimates of effect sizes (e.g. Cohen's *d*, Pearson's *r*), indicating how they were calculated

Our web collection on statistics for biologists contains articles on many of the points above.

Software and code

Policy information about availability of computer code

Data collection	GraphPad Prism 7, Microsoft Excel and BD FACsDIVA
Data analysis	GraphPad Prism 7, Microsoft Excel and FlowJo

For manuscripts utilizing custom algorithms or software that are central to the research but not yet described in published literature, software must be made available to editors/reviewers. We strongly encourage code deposition in a community repository (e.g. GitHub). See the Nature Research guidelines for submitting code & software for further information.

Data

Policy information about availability of data

All manuscripts must include a data availability statement. This statement should provide the following information, where applicable:

- Accession codes, unique identifiers, or web links for publicly available datasets
- A list of figures that have associated raw data
- A description of any restrictions on data availability

All data supporting our findings are provided within the Manuscript and Supplement.

Field-specific reporting

Please select the one below that is the best fit for your research. If you are not sure, read the appropriate sections before making your selection.

☒ Life sciences ☐ Behavioural & social sciences ☐ Ecological, evolutionary & environmental sciences

Life sciences study design

All studies must disclose on these points even when the disclosure is negative.

Sample size	No statistical method was used to determine sample size.
Data exclusions	No data were excluded from analyses
Replication	The number of times individual experiments were repeated is in the figure legend. A representative repeat was selected for publication and some replicates have been included in the supplementary materials.
Randomization	All mouse experiments were randomized. An equal number of male and female were included in each group whenever possible.
Blinding	Tumor volume measurements were blinded.

Reporting for specific materials, systems and methods

We require information from authors about some types of materials, experimental systems and methods used in many studies. Here, indicate whether each material, system or method listed is relevant to your study. If you are not sure if a list item applies to your research, read the appropriate section before selecting a response.

Materials & experimental systems

n/a	Involved in the study	
☐	☒	Antibodies
☐	☒	Eukaryotic cell lines
☒	☐	Palaeontology
☐	☒	Animals and other organisms
☒	☐	Human research participants
☒	☐	Clinical data

Methods

n/a	Involved in the study	
☒	☐	ChIP-seq
☐	☒	Flow cytometry
☒	☐	MRI-based neuroimaging

Antibodies

Antibodies used	All antibodies used, catalog number and company are listed in supplementary materials.
Validation	All antibodies used have been validated by the company selling them.

Eukaryotic cell lines

Policy information about cell lines

Cell line source(s)	HeLa R19 (Dr. E. Wimmer, State Univ. of NY at Stony Brook)<br>Jurkat 76 expressing H3.3K27M TCR and CD8 (Dr. H. Okada, UCSF, co-author)<br>CT2A (Dr. P. Fecci, Duke Univ.)<br>B16F10.9 (ATCC)<br>B16F10.9-OVA (Dr. S. Nair, Duke Univ.)<br>DIPG36 (Dr. M. Monje, Stanford Univ.)
Authentication	J76CD8+TCR+ cells were validated by flow cytometry.
Mycoplasma contamination	Cells were routinely tested for mycoplasma contamination.
Commonly misidentified lines<br>(See ICLAC register)	None

Animals and other organisms

Policy information about studies involving animals; ARRIVE guidelines recommended for reporting animal research

Laboratory animals	C57Bl6 mice transgenic for human CD155 (hCD155-tg mice) are maintained as a breeding colony (9); they were originally obtained from S. Koike (44).<br>OT-I mice were obtained from Jackson laboratories #003831<br>AAD Mice were obtained from Jackson laboratories #004191<br>AAD/hCD155 mice were obtained by breeding AAD mice with hCD155-transgenic mice.

Wild animals	No wild animals involved.
Field-collected samples	No field samples collection involved.
Ethics oversight	All mouse experiments were performed under Duke University IACUC-approved protocols.

Note that full information on the approval of the study protocol must also be provided in the manuscript.

Flow Cytometry

Plots

Confirm that:

☒ The axis labels state the marker and fluorochrome used (e.g. CD4-FITC).

☒ The axis scales are clearly visible. Include numbers along axes only for bottom left plot of group (a 'group' is an analysis of identical markers).

☒ All plots are contour plots with outliers or pseudocolor plots.

☒ A numerical value for number of cells or percentage (with statistics) is provided.

Methodology

Sample preparation	Cells were washed with FACs buffer (1XPBS + 2%FBS) and resuspended in 100 uL. Cells were Fc-blocked (TruStain, Biolegend), stained (1h) with the appropriate antibodies (Supplementary Table S2), washed and resuspended in 250uL FACs buffer to be analyzed on a BD LSRFortessaX20. Compensation and data analysis done using FlowJo.
Instrument	BD FortessaX20 (Duke Cancer Institure Flow Cytometry Core)
Software	BD FACSDiVa was used to acquire data.<br>Compensation and data analysis was performed on FlowJo (Treestar).
Cell population abundance	At least 10^5 cells were analyzed per sample in all studies.
Gating strategy	All gating strategies are shown in supplementary figures. Usual gating steps: 1. Gate out debris on FSC and SSC plot. 2. Gate on single cells (FSC-A/FSC-H). 3. Gate out dead cells.4. Gate on population of interest as using a negative sample (e.g. untreated BMDCs or human DCs), positive sample (e.g. BMDCs treated with LPS or human DCs treated with maturation cytokine cocktail). Isotype controls were used whenever relevant.

☒ Tick this box to confirm that a figure exemplifying the gating strategy is provided in the Supplementary Information.
